# Determining the Free-Carrier Fraction in 2D Perovskites Using Power Dependent Photoluminescence

**DOI:** 10.1021/acs.jpclett.6c01131

**Published:** 2026-06-16

**Authors:** Antonella Cutrupi, Marc Meléndez, Raquel Utrera-Melero, Michel Frising, Enrique Arévalo Rodríguez, Upasana Das, Ferry Prins

**Affiliations:** † Departamento de Física de la Materia Condensada, Universidad Autónoma de Madrid, 28049 Madrid, Spain; ‡ Centro de Investigacion de Física de la Materia Condensada, Universidad Autónoma de Madrid, 28049 Madrid, Spain; ¶ Instituto Nicolás Cabrera, Universidad Autónoma de Madrid, 28049 Madrid, Spain

## Abstract

Determining the nature of the optical excited state (excitons or free carriers) in nanostructured materials is crucial for device design, as optoelectronic and photovoltaic technologies require different considerations regarding the optimized excited state dynamics. Power-dependent photoluminescence is widely used to distinguish between excitons and free carriers, but the classical power-law analysis oversimplifies the underlying physics when the exponent lies between the linear (pure excitons) and quadratic (pure free carriers) limits. In this work, we present a complete study enabling a direct and quantitative analysis of the free-carrier fraction based on power-dependent peak photoluminescence and placing its analysis in the context of the Saha equation. We study Ruddlesden–Popper perovskites with varying thickness as a model system, as they cover a wide range of exciton binding energies and the full range of free carrier fractions. Our results agree with previously reported values for the exciton binding energies in these materials, confirming the reliability of this approach and providing a simple and effective tool for probing the nature of optically excited states in semiconductors with intermediate exciton binding energies. We demonstrate that our method allows probing spatial variations in the fraction of free charges near grain boundaries or edges at micrometer spatial resolution. Finally, our results highlight the importance of performing optical characterization under excitation densities relevant to realistic operating conditions, as higher fluences can artificially enhance exciton formation and distort excited-state interpretation under solar-fluence conditions.

## Graphical abstract

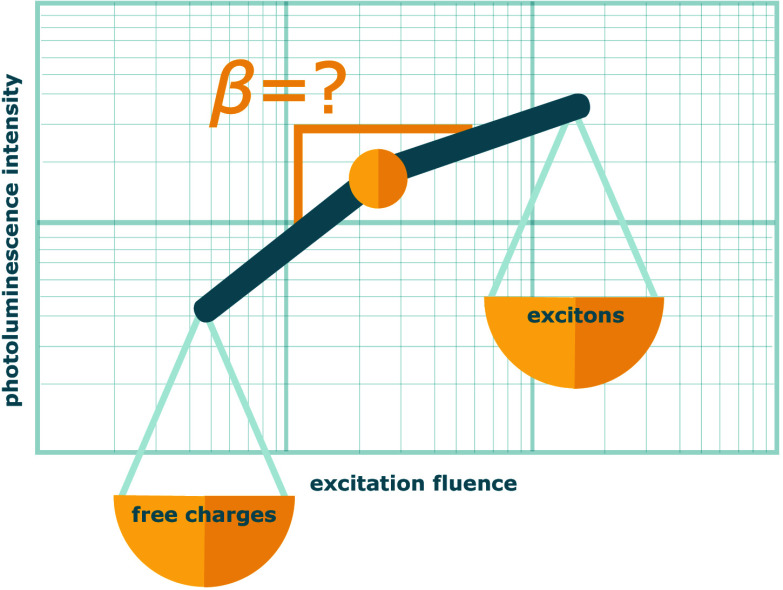

Understanding the nature of the optical excited state in nanostructured semiconductors is essential for device design.^1,2^ In quantum confined systems, strong exciton binding energies will lead to the formation of excitonic excited states where electrons and holes are bound by Coulomb forces. Excitons come in many varieties, depending on the local dielectric constants and the size and shape of the material.^3^ As a common denominator though, excitons are characterized by efficient unimolecular recombination and typically high radiative efficiency, making them appealing for light emitting applications.^4,5^ In contrast, in semiconductors with weaker quantum and dielectric confinement, the Coulomb energy is much lower and excitons readily dissociate into free charges.^6^ The optical excited state is in that case characterized by unbound electrons and holes which can be more straightforwardly collected in light harvesting devices.^7^ Consequently, different technologies require different considerations of the ideal excited state and identifying the nature of the excited state of semiconductor nanomaterials with different dimensionalities and morphologies is crucial.^8,9^

Whether the optical excited state in a system is dominated by excitons or free charges is predominantly determined by the exciton binding energy. However, materials in which the excited state is purely excitonic or purely based on free charges are only the extremes of a broader spectrum of possibilities. In the vast majority of semiconductor nanomaterials, intermediate exciton binding energies will lead to the coexistance of populations of excitons and free charges that dynamically interchange.^10–13^ This picture is further complicated by the role that excitation density plays in the formation of excitons. Following the Saha equation,^14^ the fraction of free charges *x* is related to the excitation density (*N*_exc_), the exciton binding energy (*E*_b_), and the reduced mass (μ) according to

1 $$\frac{x^{2}}{1-x}=\frac{1}{N_{exc}}{\left(\frac{2\pi \mu k_{B}T}{h^{2}}\right)}^{D/2}e^{-E_{b}/k_{B}T}$$

where *D* represents the dimensionality of the system and *T* is the temperature. This relationship highlights the importance of characterizing the optoelectronic properties of a material at technologically relevant excitation densities. The most widely employed method to identify whether the optical excited state of a material is dominated by excitons or free charges is to perform time-resolved power-dependent photoluminescence measurements (experimetal details in the SI). This type of experiment is the most reliable because the peak intensity (corresponding to the counts at *t* = 0 of the time histogram) reflects the carrier density of the system. If the photoluminescence intensity grows linearly with excitation power (*P*_out_ ∝ *P*_in_), this is indicative of first order (or unimolecular) recombination and an excitonic excited state. If the photoluminescence grows super linearly, this is a signature of second order recombination in which free electrons and holes first need to find each other before recombining. Higher excitation densities increase the recombination efficiency in that case, leading to the super linear behavior.^15^ Spectrally resolved or time-integrated experiments involving free charges can deviate from the expected power-law scaling. This deviation becomes immediately apparent when examining the lifetime trace as a function of excitation power, since the relative contribution of free charges is itself power dependent (Figure S5). An extreme case is illustrated in Figure S6, where a simulation is used to demonstrate how the peak intensity and the integrated intensity give a different power law exponent. The latter does not reflect the actual type of carriers present in the system.

Using power law fits $\left(P_{out}\propto P_{in}^{\beta }\right)$ to describe the excitonic nature is common practice,^16^ with β = 1 indicating excitons, β = 2 free charges, and intermediate values often interpreted as a mixture of both.^17^ However, questions remain about the meaning of such intermediate β values and whether they can be translated to a physically more meaningful value of a free carrier fraction. Even more importantly though, as discussed above, the carrier density itself influences the free carrier fraction, suggesting that a power law relation is an oversimplification of the underlying physics. At low fluencies, the power dependence should represent the quadratic scaling of free charge carriers while at higher fluencies the scaling should become linear as excitons start dominating the excited state population. Importantly, the observed linear scaling at high fluences also excludes the possibility of Meitner-Auger recombination or effects of photobrightning, which would result in nonlinear scaling at high powers.^18,19^ A previous study, Wang et al.^20^ introduced a density-resolved spectroscopy method to demonstrate the coexistence of excitons and free carriers over a wide density range in the three-dimensional perovskite CH_3_NH_3_PbI_3_. In that work, a three-dimensional form of the Saha equation was employed to extract the exciton binding energy. Therefore, careful consideration of the excitation regime in which the power dependence is measured is essential for accurately determining the nature of the excited states in a material at a given fluence.

Here, we present a complete study using power dependent photoluminescence measurements that not only allows to correct for the excitation density, but moreover allows to directly determine the free carrier fraction *x* from such measurements. For this, we study Ruddlesden–Popper perovskites with varying thickness *n*, including a series of butylammonium (BA)-based methylammonium lead iodide (BA)_2_(MA)_*n*−1_Pb_*n*_I_3*n*+1_ (*n* = 1, 2, 3, 4, 5), and a series of phenethylammonium (PEA)-based formamidinium lead iodide (PEA)_2_(FA)_*n*−1_Pb_*n*_I_3*n*+1_ (*n* = 1, 2). This family covers a wide range of exciton binding energies,^2,21^ comprising the full range of free carrier fractions, from pure excitonic to pure free charge based excited states.^13,22^ Our results from power dependent photoluminescence compare favorably to more elaborate techniques using microwave conductivity.^23^In addition, we demonstrate how this method can be used to investigate variations in the fraction of free charges near grain boundaries or edges, where edge states may promote exciton dissociation in 2D perovskites, as suggested by previous studies.^24,25^

We prepare a series of (BA)_2_(MA)_*n*−1_Pb_*n*_I_3*n*+1_ Ruddlesden–Popper perovskites, in which butylammonium (BA) is the long organic cation that separates the inorganic layers of corner sharing lead iodide octahedra, methylammonium (MA) is the small interstitial cation, and *n* is the number of octahedra that make up the thickness of the inorganic layer (Figure 1(a)).^26^ Millimeter-sized flakes of (BA)_2_(MA)_*n*−1_Pb_*n*_I_3*n*+1_ (Figure 1(b)) are synthesized via solution-based methods^21^ (more details in the SI) and exfoliated onto transparent substrates for optical characterization. The photoluminescence (PL) spectra exhibit a clear redshift as *n* increases (Figure 1(c)), as the increase in the thickness of the inorganic layers reduces the quantum and dielectric confinement effects, leading to a decrease in the bandgap energy and thus shifting the emission peak to the near-infrared region.^27–29^

**Figure 1:**
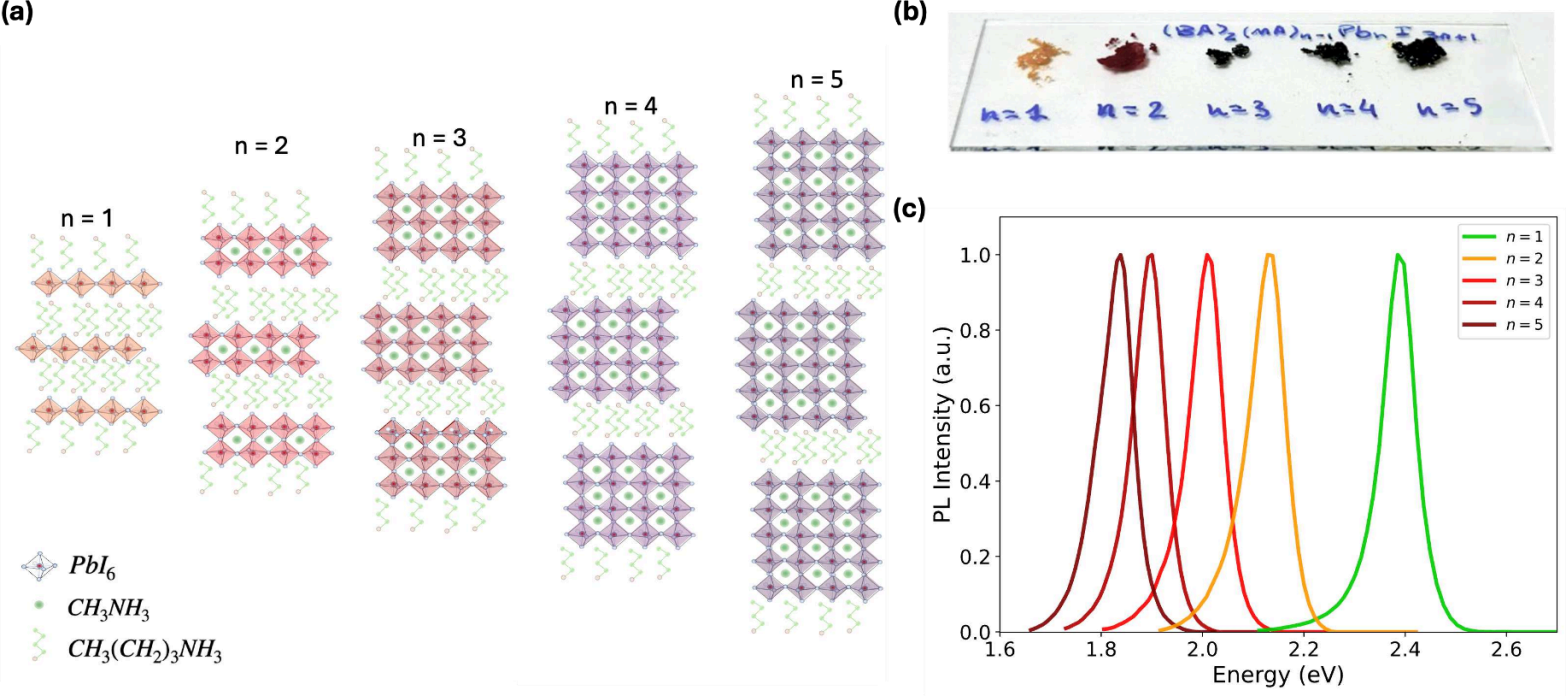
(a) Schematic structure of layered two-dimensional (2D) Ruddlesden–Popper perovskites. (b) Millimeter-sized flakes of (BA)_2_(MA)_*n*−1_Pb_*n*_I_3*n*+1_ for different values of *n*. (c) Normalized photoluminescence spectra of different samples.

To determine the excitonic or free-carrier nature of the photoexcited states, we perform time-resolved photoluminescence measurements (TRPL) at different excitation powers and extract maximum photoluminescence intensity (*t* = 0). All measurements were performed at room temperature using time-correlated single-photon counting with a pulsed laser diode and a defocused laser spot of a few microns in diameter. Laser power was controlled automatically with a home-built motorized neutral-density filter wheel. Full experimental details are presented in the SI. In Figure 2(a–c), we present the excitation density dependence of the *t* = 0 photoluminescence intensity of *n* = 1, 3, 5 (measurements for remaining *n* are reported in the SI, Figure S4). With increasing *n*, a clear transition from linear to super linear scaling with the excitation density is observed. Performing a power law fit, we can extract the exponent β, as shown with dashed lines for *n* = 1, 3, 5 in Figure 2(a–c). At lower *n*, the β exponent is close to 1, indicating that the emission is primarily excitonic in nature, consistent with strong quantum confinement and large excitonic binding energy.^30^ For larger *n*, β increases progressively, indicating a shift to bimolecular recombination that is increasingly dominated by free charges. The observed evolution of β as a function of *n* (Figure 2(d)) is in good agreement with previous studies on the same family of Ruddlesden–Popper perovskites.^17^

**Figure 2:**
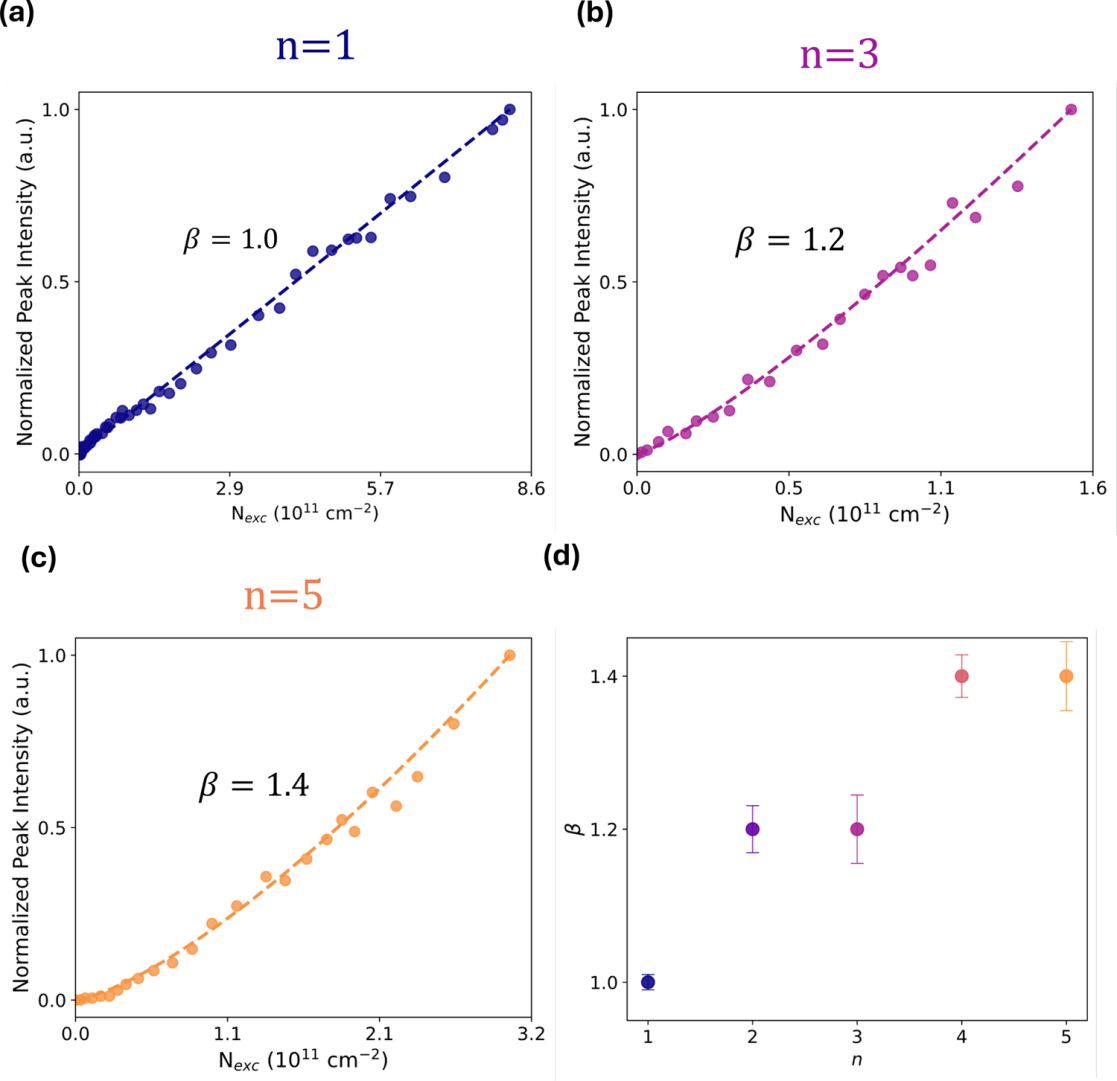
(a–c) Power law fit to the time-resolved photoluminescence experiments for *n* = 1, 3, 5. The dots represent the experimental data, while the dashed lines correspond to the power-law fits *I* ∝ *P* ^β^. (d) The parameter β denotes the power law exponent, which characterizes the nature of the excited states. Excitonic (β = 1) in the case of *n* = 1, mixture (1 < β < 2) for *n* = 3 and *n* = 5.

While the variation in the β exponent gives a qualitative indication of the nature of the excited state, it is important to emphasize that the power law fit is an oversimplification of the expected behavior that ignores the dependence of the free carrier fraction on the excitation density *N*_exc_.^20^ Indeed, plotting the power dependence data on a log–log scale (see Figure 3(a)) we observe that the slope varies with the excitation density particularly for intermediate values of *n*. This variation reflects a transition from a super linear behavior at low densities to a linear regime at high densities, highlighting the dependence of the power-law exponent (β) on the excitation density (Figure S4 in the SI).

**Figure 3:**
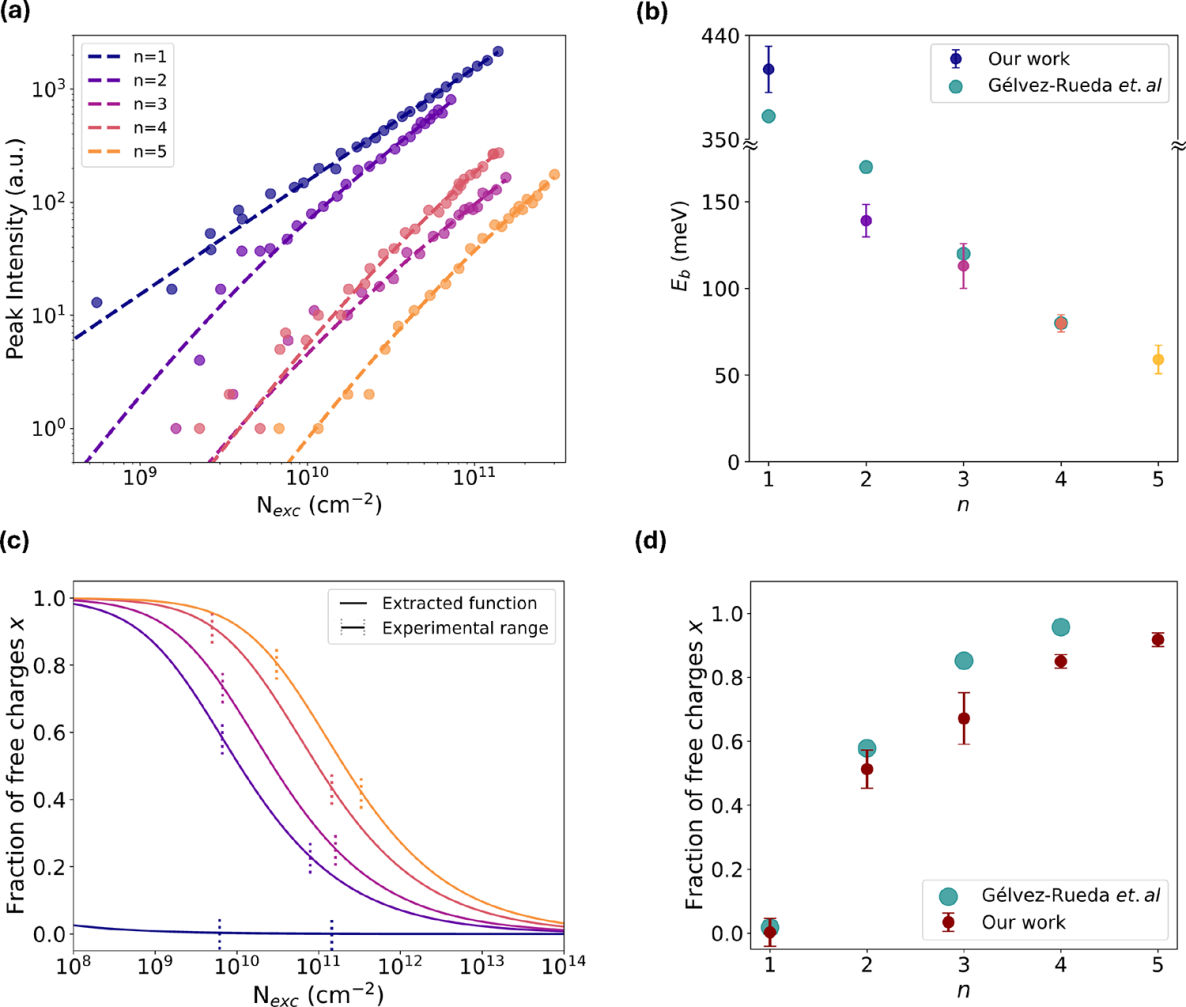
(a) Fitting of eq 5 to the time-resolved photoluminescence experiments for *n* = 1, 2, 3, 4, 5. The dots represent the experimental data, while the dashed lines correspond to the fits. (b) Exciton binding energies extracted from the Saha prefactor *A* (eq 3, values are listed in Table S1 in the SI). (c) Fraction of free charges *x* (eq 4) calculated using the fit results, extended over the range 10^8^ cm^–2^ < *N*_exc_ < 10^14^ cm^–2^. Vertical dashed lines show the experimental range. (d) Extracted values of the fraction of free charges at solar fluence (*N*_exc_ = 10^10^ cm^–2^, brown dots). Exciton binding energies and fraction of free charges are compared with those reported by Gélvez-Rueda et al. in their study.^23^

Using the model that can account for the excitation-density dependence of the free-carrier fraction,^20^ we take the two-dimensional form of the Saha equation (eq 1). This choice is justified by the fact that the Ruddlesden–Popper perovskite series considered in this study consists of 2D or quasi-2D systems, with

2 $$\frac{x^{2}}{1-x}=\frac{1}{N_{exc}}\frac{2\pi \mu k_{B}T}{h^{2}}e^{-E_{b}/k_{B}T}$$

where the fraction *x* of the population that represents excitations in the form of pairs of free charges depends on the concentration (number density) of excitations *N*_exc_, μ is the reduced mass of the exciton (more details in the SI), and *E*_b_ is the excitation binding energy (reported values are listed in Table S1 in the SI).^31^ For simplicity, we can define a Saha prefactor

3 $$A=\frac{2\pi \mu k_{B}T}{h^{2}}e^{-E_{b}/k_{B}T}$$

and taking $ñ=N_{exc}/A$ and solving eq 2 for *x*. We obtain the fraction of free carriers *x* expressed as

4 $$x=\frac{1}{2ñ}\left(\sqrt{4ñ+1}-1\right)$$

Considering the rate equation $\frac{dN}{dt}=-\rho x^{2}N^{2}+\nu (1-x)N$ (more details in the SI), the photons generated by excitonic recombination increase linearly with concentration, whereas those emitted by free charge recombination scale quadratically within a short time window Δ*t*. Therefore, the photon emission intensity for a carrier concentration ñ can be expressed as follows:

5 $$E=\left(\rho x^{2}{ñ}^{2}+\nu \left(1-x\right)ñ\right)\Delta t=\left(\rho +\nu \right)\left(ñ+\frac{1}{2}-\sqrt{ñ+\frac{1}{4}}\right)\Delta t$$

yielding an expression for the photoluminescence intensity *E* that explicitly captures the dependence of the free-carrier fraction on the excitation density. Then, we can define the slope of our function, β(ñ) , as the partial derivative of ln(ñ) :

$$\beta \left(ñ\right)=\frac{\partial }{\partial \left(\ln\left(ñ\right)\right)}\;\ln\left(\frac{E}{\left(\rho +\nu \right)\Delta t}\right)=\frac{ñ-\frac{ñ}{2\sqrt{ñ+\frac{1}{4}}}}{ñ+\frac{1}{2}-\sqrt{ñ+\frac{1}{4}}}$$

Fitting the data of Figure 2 to eq 5 and representing it on a log–log scale (Figure 3(a)) allows us to extract (ρ + ν) and Saha prefactor *A* (eq 3). From these parameters, the exciton binding energy (*E*_b_) of our materials can be determined (Figure 3(b)), showing the expected gradual evolution toward lower exciton binding energies for increasing inorganic layer thickness.^32,33^

Using the extracted values for the exciton binding energies, we extrapolate the fraction of free charges as a function of the excition density following eq 4 (see Figure 3(c)). This allows us to calculate the corresponding free-charge fraction at solar fluence (*N*_exc_ = 10^10^ cm^–2^), plotted in Figure 3(d) (brown dots). Importantly, our results are in good agreement with the free-charge fraction obtained via more elaborate microwave conductivity measurements of Gélvez-Rueda et al.^23^ on the same set of RP perovskites.

An additional advantage of the power dependent analysis is the possibility to determine the free charge carrier fraction with micrometer spatial resolution. As previous reports have suggested, edge states in two-dimensional perovskites can promote exciton dissociation and locally enhance the fraction of free charge carriers.^24,34^ If exciton dissociation occurs at grain boundaries or flake edges, a reduced effective exciton binding energy would be observed with respect to the theoretical value estimated for homogeneous regions of the material. To investigate this scenario, we perform power dependent TRPL experiments on a spin-coated film of (PEA)_2_PbI_4_ (*n* = 1) perovskite. We measure the exciton fraction between the homogeneous regions (dark blue marker in Figure 4(a)) and grain boundaries (yellow marker). As expected, in the homogeneous regions we recover exciton binding energy consistent with previously reported values for the PEA series^35^ (see Figure S7 in the SI). In contrast, we observe an effective reduction of the exciton binding energy at grain boundaries (Figure 4(b)) and can extract the corresponding increase in the fraction of free charges (Figure 4(c)) using eq 4. In the Supporting Information (see Figure S8) we present additional measurements on an exfoliated flake of (PEA)_2_FAPb_6_I_7_ (*n* = 2) perovskite, observing the same trend when comparing the central region with the edges of the flake.

**Figure 4:**
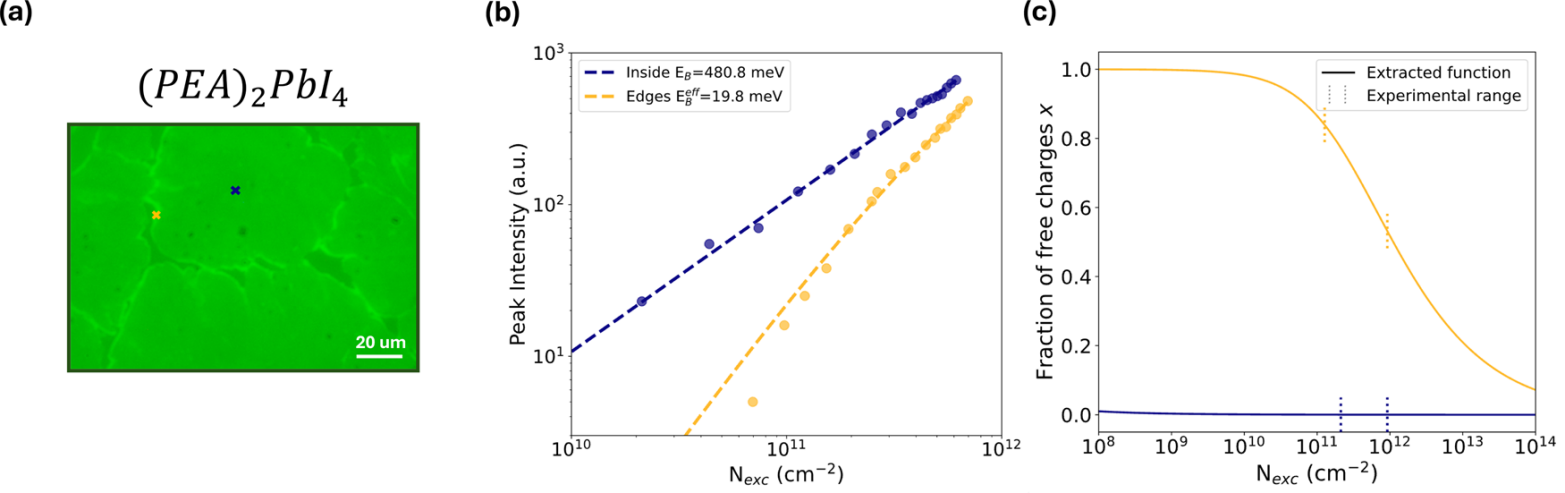
(a) Reflectivity image of PEA_2_PbI_4_ obtained by spin-coating. The marker (*x*) indicates the two regions where the time-resolved photoluminescence (TRPL) experiments were performed. (b) Fitting of eq 5 to the TRPL experiments. The dots represent the experimental data, while the dashed lines correspond to the fits. (c) Fraction of free charges *x* (eq 4) calculated using the fit results, extended over the range 10^8^ cm^–2^ < *N*_exc_ < 10^14^ cm^–2^.

In conclusion, we have presented a model that enables direct quantitative analysis of the free-carrier fraction using commonly employed power-dependent fluorescence measurements. A key advantage of this model is that it explicitly incorporates the dependence of the free-carrier fraction on excitation density, allowing accurate extrapolation across a wide range of excitation fluencies. Applying this approach to a family of Ruddlesden–Popper layered perovskites, we find that the extracted exciton binding energies and free-carrier fractions closely match values obtained from more sophisticated techniques, such as microwave conductivity.^23^ This agreement highlights the promise of our model as a simple and effective tool for probing the nature of optically excited states in semiconductors with intermediate exciton binding energies.

In this respect, it is important to compare our method to alternative approaches that extract the free carrier fraction directly from the photoluminescence decay dynamics, as proposed by Spitha et al.^35^ using a kinetic model. Crucially though, in the absence of detailed information on the relative photoluminescence quantum yield of exciton and free charge recombination, such kinetic models become considerably less reliable for estimating the free-carrier fraction. Our approach based on power dependence of the peak photoluminescence circumvents this issue by extracting the free carrier fraction from the relative slopes. A detailed analysis of the decay dynamics is provided in the Supporting Information (see Figures S9 and S10).

Finally, we emphasize the importance of conducting optical characterization at excitation densities relevant to the intended operating conditions. The high excitation levels often used in optical spectroscopy and microscopy can artificially enhance exciton formation, potentially misrepresenting the behavior of the excited state under realistic solar-fluence conditions.

## Supplementary Material



## Data Availability

The data sets used in this study are openly accessible via the following link 10.21950/MPEY3Z. The code used for data processing and analysis is available at https://github.com/phond-uam/Power_Dependent_Photoluminescence.

## Notes

The materials used for sample synthesis, sample preparation procedures, and experimental details for X-ray diffraction, photoluminescence (PL) spectra, PL lifetime measurements, and power-dependent PL measurements using time-correlated single photon counting (TCSPC); additional figures and discussion of results complementary to the main text, as well as details of the theoretical model, simulations, and fitting procedures (PDF)
