# Supplementary material for: Determining the Free-Carrier Fraction in 2D Perovskites Using Power Dependent Photoluminescence
Source: J Phys Chem Lett. 2026 Jun 16;17(26):7336–42. doi: 10.1021/acs.jpclett.6c01131 (PMC13339772; doi:10.1021/acs.jpclett.6c01131)
Supplement: Supplementary file 1 [file jz6c01131_si_001.pdf]

Supporting Information: Determining the Free-Carrier Fraction in 2D Perovskites using Power Dependent Photoluminescence.

Antonella Cutrupi,[†,‡,¶] Marc Meléndez,[†,‡] Raquel Utrera-Melero,[†,‡] Michel Frising,[†,‡] Enrique Arévalo Rodríguez,[†,‡,¶] Upasana Das,[†,‡,¶] and Ferry Prins[†,‡,¶]

†*Departamento de Física de la Materia Condensada, Universidad Autónoma de Madrid, 28049 Madrid, Spain*

‡*Centro de Investigacion de Física de la Materia Condensada, Universidad Autónoma de Madrid, 28049 Madrid, Spain*

¶*Instituto Nicolás Cabrera, Universidad Autónoma de Madrid, 28049 Madrid, Spain*

Materials

Microcrystals of Ruddlesden–Popper Layered Perovskites.

PbO powder and 50% aqueous hypophosphorous acid solution (H_3PO_2) were purchased from Sigma Aldrich. Methylammonium iodine (CH_3NH_3I) and n-Buthylammonium iodine (n-$CH_3(CH_2)_3NH_2HI$) were purchased from Greatcell Solar Materials. Hydroiodic acid (HI, 57% w/w) was purchased from TCI Chemicals. All the reagents were used without further purification.

Thin films by spin-coating and drop-casting of Ruddlesden–Popper Layered Perovskites.

Phenetylammonioum iodide ($C_6H_5(CH_2)_2NH_3I$), Formamidinium iodide ($CH(NH_2)_2I$), Lead iodide (PbI_2), Dimetilformammide (C_3H_7NO), and γ-butyrolactone ($C_4H_6O_2$), were purchased from Sigma Aldrich and used as received.

Sample preparation

Ruddelson Popper perovskites (RPs) $(BA)_2(MA)_{n-1}Pb_nI_{3n+1}$ microcrystals.

$(BA)_2PbI_4$ ($n = 1$)

PbO powder (223 mg, 1 mmol) was dissolved in a mixture of 57% w/w aqueous HI solution (1 mL) and 50% aqueous H_3PO_2 (170 μL). This mixture was heated to boiling under constant magnetic stirring, which formed a yellow solution. A second solution of n-$CH_3(CH_2)_3NH_2HI$ salt (201 mg, 1 mmol) in HI 57 % w/w (0.5 mL) was prepared. Addition of the n-$CH_3(CH_2)_3NH_3HI$ solution to the initial PbI_2 solution, produces a black precipitate, which subsequently dissolved under heating the combined solution to boiling. The stirring was stopped, and the solution was left to cool to room temperature for around 1 hour giving rise to orange rectangular-shaped plates. The crystals were isolated by suction filtration. IR (ATR): 3380br, 3162m, 3053m, 3014m, 2956m, 2926m, 2868m, 2471w, 2385w, 1615m, 1568s, 1463s, 1389m, 1374m, 1152m, 1065s, 1037s, 1000m, 919s, 910w, 796vw, 781w, 744w, 734m, 475m.

$(BA)_2(MA)Pb_2I_7$ (n = 2)

PbO powder (223 mg, 1 mmol) was dissolved in a mixture of 57% w/w aqueous HI solution (1 mL) and 50% aqueous H_3PO_2 (170 μL). This mixture was heated to boiling under constant magnetic stirring, which formed a yellow solution. CH_3NH_3I (79.5 mg, 0.5 mmol) solid

was added to the hot yellow solution, initially caused the precipitation of a black powder, which rapidly redissolved under stirring to afford a clear bright yellow solution. A second solution of n-$CH_3(CH_2)_3$ NH_2HI salt (141, 0.7 mmol) in HI 57% w/w (0.5 mL) was prepared. Addition of the n-$CH_3(CH_2)_3NH_3HI$ solution to the initial PbI_2 solution, produces a black precipitate, which subsequently dissolved under heating the combined solution to boiling. The stirring was stopped, and the solution was left to cool to room temperature for around 1 hour giving rise to cherry red rectangular-shaped plates. The crystals were isolated by suction filtration. IR (ATR): 3364br, 3175w, 2956m, 2926m, 2868m, 1619m, 1572m, 1458s, 1378w, 1146s, 1065m, 1028w, 1000m, 967w, 901m, 788w, 744w, 734m, 475m.

$(BA)_2(MA)_2Pb_3I_{10}$(n = 3)

PbO powder (223 mg, 1 mmol) was dissolved in a mixture of 57% w/w aqueous HI solution (1 mL) and 50% aqueous H_3P0_2 (170 μL). This mixture was heated to boiling under constant magnetic stirring, which formed a yellow solution. CH_3NH_3I (105.9 mg, 0.67 mmol) solid was added to the hot yellow solution, initially caused the precipitation of a black powder, which rapidly redissolved under stirring to afford a clear bright yellow solution. A second solution of n-$CH_3(CH_2)_3NH_2HI$ salt (66.5, 0.33 mmol) in HI 57% w/w (0.5 mL) was prepared. Addition of the n-$CH_3(CH_2)_3NH_3HI$ solution to the initial PbI_2 solution, produces a black precipitate, which subsequently dissolved under heating the combined solution to boiling. The stirring was stopped, and the solution was left to cool to room temperature for around 1 hour giving rise to deep-red/purple rectangular-shaped plates. The crystals were isolated by suction filtration. IR (ATR): 3362s, 3156m, 2956w, 2926w, 2352w, 2090m, 1989w, 1605s, 1452m, 1378w, 1137m, 1065w, 1020w, 981w, 952w, 901m, 792w, 744w, 734w, 479m, 413m.

$(BA)_2(MA)_3Pb_4I_{13}$(n = 4)

PbO powder (223 mg, 1 mmol) was dissolved in a mixture of 57% w/w aqueous HI solution (1 mL) and 50% aqueous H_3P0_2 (170 μL). This mixture was heated to boiling under constant magnetic stirring, which formed a yellow solution. CH_3NH_3I (119 mg, 0.75 mmol) solid was added to the hot yellow solution, initially caused the precipitation of a black powder, which

rapidly redissolved under stirring to afford a clear bright yellow solution. A second solution of n-$CH_3(CH_2)_3NH_2HI$ salt (50.3 mg, 0.25 mmol) in HI 57% w/w (0.5 mL) was prepared. Addition of the n-$CH_3(CH_2)_3NH_2HI$ solution to the initial PbI_2 solution, produces a black precipitate, which subsequently dissolved under heating the combined solution to boiling. The stirring was stopped, and the solution was left to cool to room temperature for around 1 hour giving rise to black crystals. The crystals were isolated by suction filtration. IR (ATR): 3352br, 2459w, 2368w, 2098m, 1985w, 1605s, 1448m, 1146m, 1104s, 891m, 790m, 736m, 477m, 411m.

$(BA)_2(MA)_4Pb_5I_{16}$(n = 5)

PbO powder (223 mg, 1 mmol) was dissolved in a mixture of 57% w/w aqueous HI solution (1 mL) and 50% aqueous H_3P0_2 (170 μL). This mixture was heated to boiling under constant magnetic stirring, which formed a yellow solution. CH_3NH_3I (127.9 mg, 0.8 mmol) solid was added to the hot yellow solution, initially caused the precipitation of a black powder, which rapidly redissolved under stirring to afford a clear bright yellow solution. A second solution of n-$CH_3(CH_2)_3NH_2HI$ salt (40.5 mg, 0.2 mmol) in HI 57% w/w (0.5 mL) was prepared. Addition of the n- $CH_3(CH_2)_3NH_2HI$ solution to the initial PbI_2 solution, produces a black precipitate, which subsequently dissolved under heating the combined solution to boiling. The stirring was stopped, and the solution was left to cool to room temperature for around 1 hour giving rise to black crystals. The crystals were isolated by suction filtration. IR (ATR): 3366br, 2953br, 2372s, 2119m, 1619s, 1561m, 1458m, 1389w, 1146m, 985s, 895s, 788w, 732m, 479s.

$(PEA)_2PbI_4$ (n = 1)

In short, the precursor salts $C_6H_5(CH_2)_2NH_3I$ and PbI_2 were mixed in a stoichiometric ratio of 2:1 and dissolved dissolved in 1mL of Dimetilformammide. It was then spin coated, on heated substrate, at 1000 rpm/s for 40 seconds and heated at 70°C for 10 minutes.

$(PEA)_2FAPb_6I_7$ (n = 2)

In short, the precursor salts $C_6H_5(CH_2)_2NH_3I$, PbI_2, and $(CH(NH_2)_2I)$ were mixed in a stoichiometric ratio of 2:1:1 and dissolved in 1 mL of γ-butyrolactone. The solution was heated and maintained at 70°C until it reached supersaturation. It was then cooled to room temperature and drop-cast onto a glass slide. Nitto tape was used to transfer microsized flakes for optical characterization.

Experimetal details

X-ray Diffraction (XRD)

X-ray diffraction measurements for microflakes of $(BA)_2(MA)_{n-1}Pb_nI_{3n+1}$ ($n = 1, 2, 3, 4, 5$) and drop-cast $(PEA)_2FAPb_2I_7$ (n=2) were performed in a Bruker D8 Discover with a $\theta/2\theta$ geometry. A primary Ge 111 Johansson curved Monocromator,to obtain the Cu-$K\alpha_1$ radiation and a 1-D LYNXEYE XE-T detector to eliminate the fluorescence and the Kß radiation were used. For the spin-coated $(PEA)_2PbI_4$ (n=1), a X'Pert PRO from Panalytical with $\theta/2\theta$ geometry was used. Optics and diffracted beam detection assembly for parallel beam, with two collimators and multipurpose sample holder platform, MPSS with secondary flat crystal monochromator and Xenon detector were used. In all measurements, the range was set from 3° to 60°.

PL Spectra Measurements

PL measurements were performed with the exfoliated sample mounted on an inverted microscope (Nikon Eclipse Ti-U). The excitation source was a 375 nm pulsed laser diode (PicoQuant LDH-D-C-375, PDL 800-D). The output beam was directed to an inverted optical microscope (Nikon Eclipse Ti-U) and the excitation laser was filtered out using a dichroic beamsplitter (SemRock FF376-Di01-25X36). The fluorescence beam was focused into the spectrograph (Princeton Instruments SpectraPro HRS-300 imaging spectrograph), diffracted by a grating of density 300 g/mm, blaze of 500 nm, and slit width sets to 100 μm.

PL Lifetime Measurements

Samples were excited with a pulsed 375 nm laser (PicoQuant LDH-D-C-375, driven by a PDL 800-D controller, 1 MHz), which was focused down to a near-diffraction-limited spot. The PL was collected using 60x objective (Nikon CFI S Plan Fluor, NA = 0.7) , and the excitation laser was filtered out using a dichroic beamsplitter (SemRock FF376-Di01-25X36). The fluorescence was then focused onto an avalanche photodiode (APD, Micro Photo Devices PDM, 20 x 20 μm^2 detector size) using a low magnification objective (Nikon Plan Fluor 10x). The laser and APD are synchronized using an electronic time-correlated single photon counting board (PicoHarp 300).

Power dependent photoluminescence using time-correlated single photon counting (TCSPC)

Power dependence PL spectroscopy was performed using an electronic time-correlated single photon counting system (TCSPC), PicoHarp 300. The excitation source was a 375 nm pulsed laser diode (PicoQuant LDH-D-C-375, PDL 800-D), and it was filtered with a dichroic beamsplitter (SemRock FF376-Di01-25X36). A 60x objective (Nikon CFI S Plan Fluor, NA = 0.7) was used to focus down the laser beam to a spot with a full width at half maximum (FWHM) of approximately 3/4 μm. The excitation power is controlled through the automatization (Arduino controlled with a custom Python script) of a neutral density filter wheel (Thorlabs NDC-100C-4M-A), allowing us to achieve sub-nanowatt resolution in excitation control with a continuous change of the excitation power. The emitted PL signal is focused on a single photon detecting avalanche photodiode (APD, Micro Photon Devices PDM, 20×20 μm^2 detector) using a low magnification objective (Nikon Plan Fluor 10x). The experimental data were collected using a custom Python script, which recorded a TCSPC histogram for each power value (USB Power Meter - Thorlabs PM16-122).

Experimental results

Figure S1 (a) shows the XRD patterns of the BA–MA series and (b) those of the PEA-FA series. These results confirm the formation of pure phase in the analyzed samples, in agreement with previous reports.[1–5]

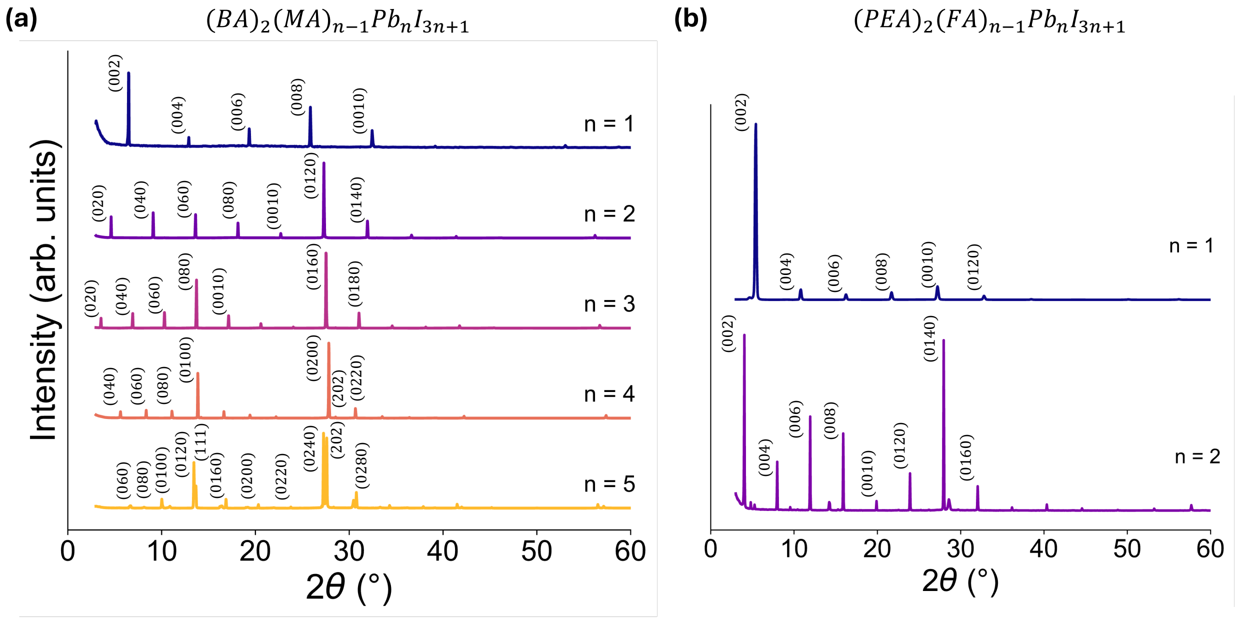

Figure S1: X-ray diffraction patterns (Cu $K\alpha_1$) of the (a) $(BA)_2(MA)_{n-1}Pb_nI_{3n+1}$ perovskites[1,2] and (b) $(PEA)_2(FA)_{n-1}Pb_nI_{3n+1}$ perovskites.[3–5]

Figure S2 provides an overview of the experimental workflow reporting synthesis steps used to obtain the layered material.[6] Optical images of the exfoliated flakes corresponding to $n = 1, 2, 3, 4, 5$ are reported in Figure S3 (a). The lifetime traces for $n = 1, 2, 3, 4, 5$, measured under the same excitation fluence of 40 μJcm^{-2} (b), show that the effective PL lifetimes vs n (c) increases consistently with the number of layers, as reported previously.[7] Figure S4 shows the standard power-dependence analysis using power law fits to the time-resolved photoluminescence (TRPL) experiments. The power law exponent β equals unity ($\beta = 1$) for $n = 1$, as corresponds to the excitonic system, and it increases with the number of layers ($1 < \beta < 2$ for $n > 1$), as expected.

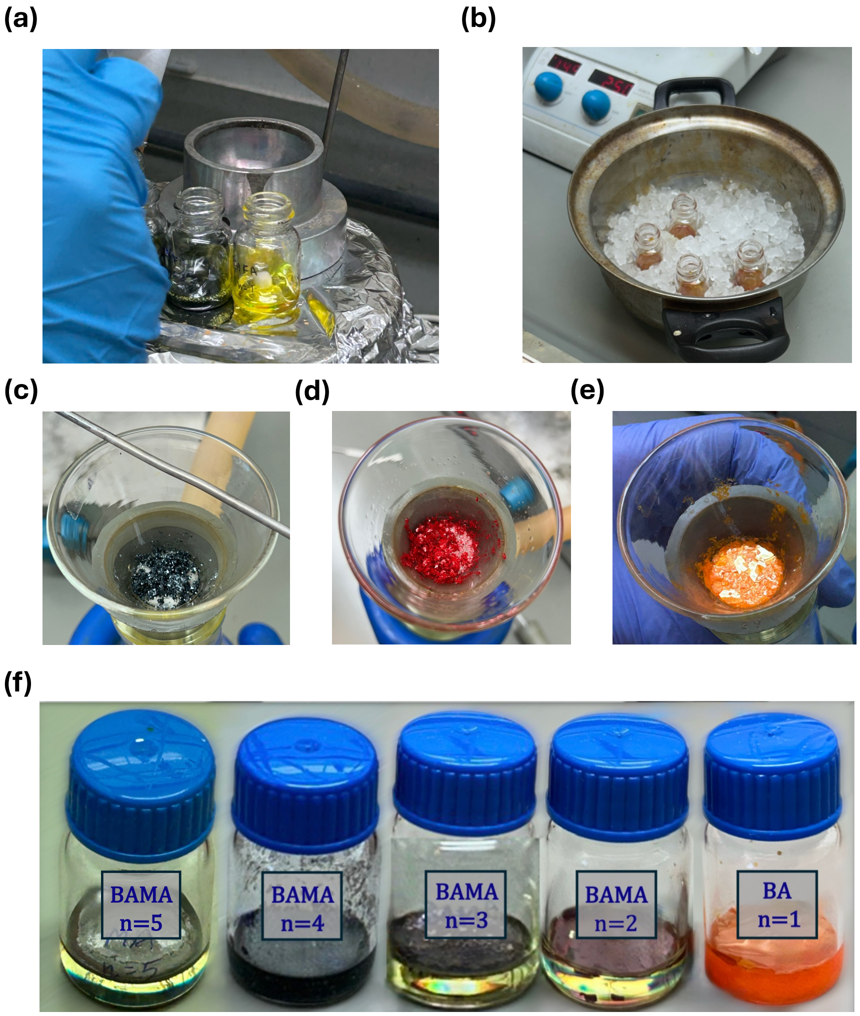

Figure S2: Schematic representation of the synthesis steps (a) Heating reaction process, (b) BA salt solutions in bath-ice, (c) crystals in solution during crystallization process, (d) filtered crystals of n=3 perovskite, (e) filtered crystals of n=2 perovskite, and (f) filtered crystals of n=1 perovskite.[6]

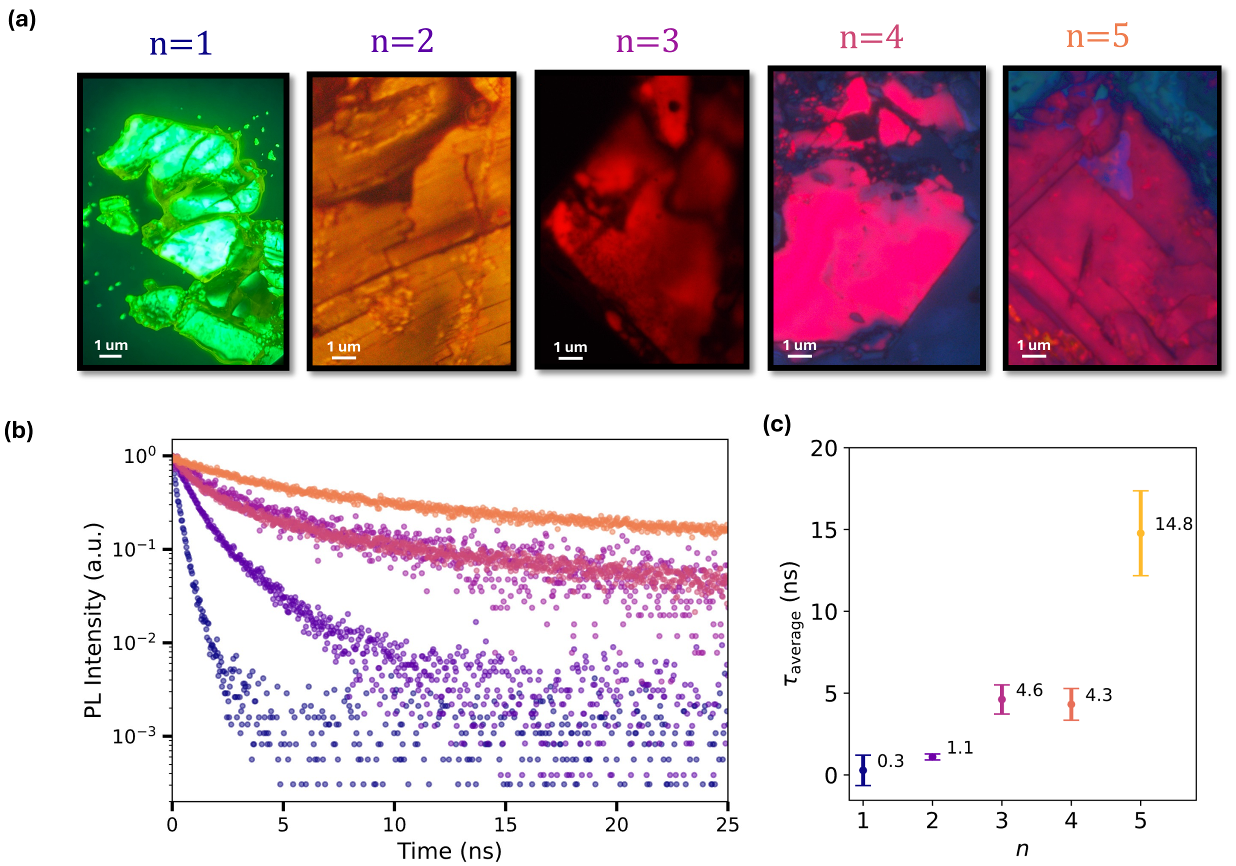

Figure S3: (a) Optical images of the exfoliated flakes for $n = 1, 2, 3, 4, 5$. (b) Time-resolved PL decay for each sample. (c) Average PL lifetime as a function of n. Please note that for n=1, the value is smaller than the statistical error.

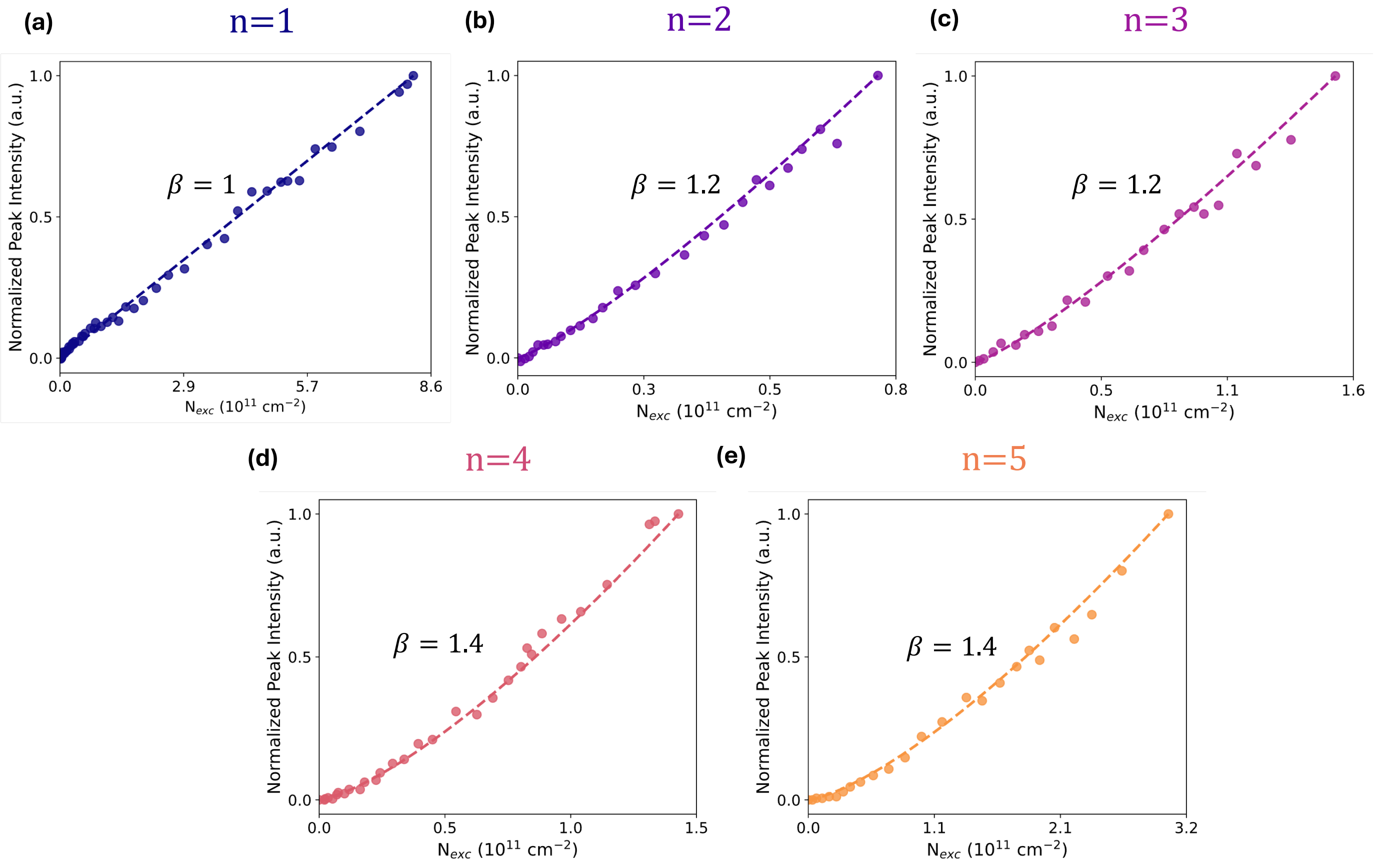

Figure S4: (a-e) Power-law fits to the time-resolved photoluminescence (TRPL) experiments for all measured samples with n = 1,2,3,4,5. The dots represent the experimental PL decay data, while the dashed lines correspond to the fits of the form $I \propto P^{\beta}$.

Figure S5 shows the TRPL dataset performed on BA-MA RP perovskites series for different n from which peak intensities (counts at t=0) were extracted across power ranges. It is evident that the lifetime is independent of excitation density for $n = 1$ (Figure S5 (a)), consistent with a purely excitonic regime, whereas a clear power dependence emerges as n increases (Figure S5 (b-e)). This reflects the growing contribution of free charge carriers, whose relative population depends on excitation density (see Equation 1). Figure S6 illustrates the fact that the scaling exhibited by the peak photoluminescence PL_0 may differ from the behavior of the time-integrated photoluminescence. In the figure, a diffusing population of free charges displays a quadratic dependence of PL_0 on the excitation density N_{exc}, whereas the time-integrated PL depends on N_{exc} linearly. The numerical simulation

considered an initial concentration field of free charges, $n(x,t)$, which obeyed the following partial differential equation law for diffusion and pair recombination:

$$\frac{\partial n}{\partial t} = D\frac{\partial^2 n}{\partial x^2} - \rho n^2, \tag{1}$$

where D stands for the diffusion coefficient. At each time and position, the photoluminescence was calculated as the number of recombinations multiplied by the quantum yield q_f, that is, $q_f\rho(n(x,t))^2$. If we set the quantum yield equal to unity $q_f = 1$, charges diffuse until they eventually recombine with an opposite charge and give rise to a photon. This implies that the time-integrated photon count equals the number of charge pairs created initially, and thus depends linearly on the incident laser power. However, the number of photons generated at time $t = 0$ at the laser focus (PL_0) is still proportional to the square of the initial population, and therefore increases quadratically with the power. This remains true as long as the population is dominated by free charges. As the density increases, the Saha equation predicts that the population will end up having a higher and higher proportion of excitons and the PL_0 vs power trend will become linear. Figure S7 shows the TRPL experiments performed on PEA-FA RP perovskites series with n=1 and 2. The extracted exciton binding energies are in good agreement with values reported for this class of materials,[8] confirming the expected decrease with increasing number of layers.

Figure S8 reports the power dependent TRPL experiments on the exfoliated flake of single crystal of $(PEA)_2FAPb_6I_7$ (n=2) perovskite (a), the fitting using the Saha correction (b), the extracted exciton binding energies inside the flake and at the edges, and the corresponding increase in the fraction of free charges (c).

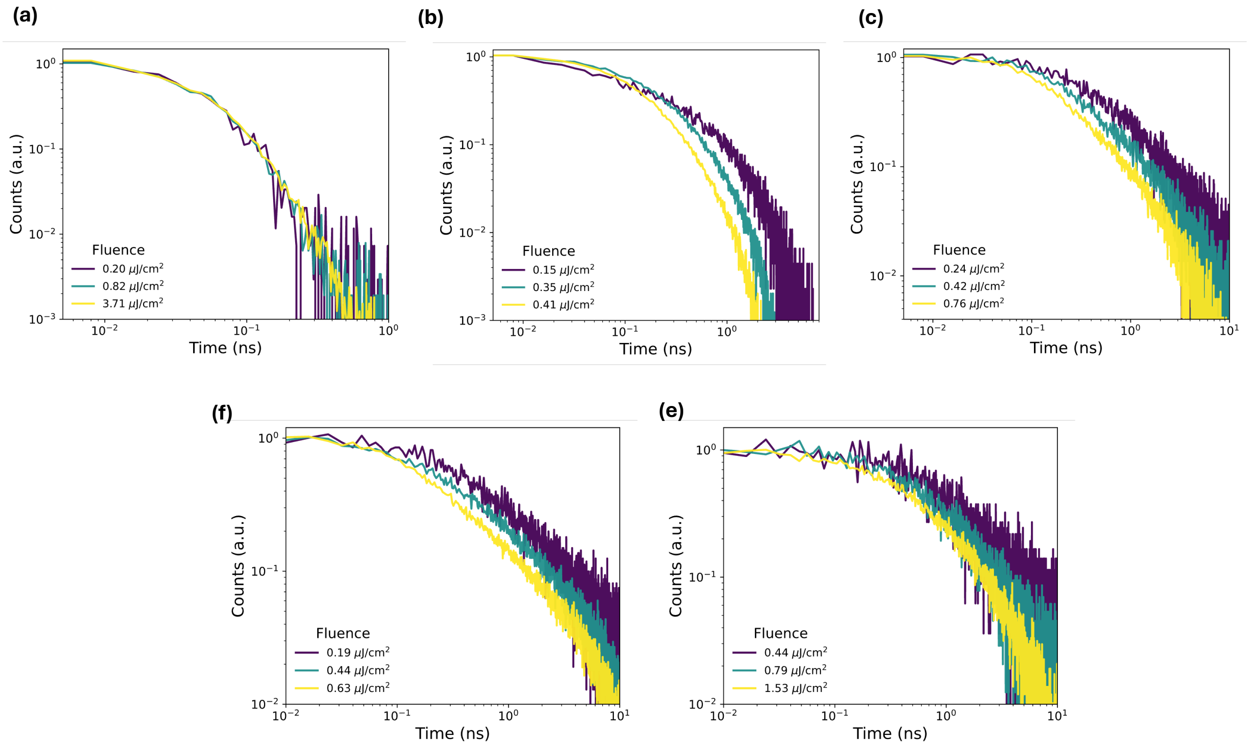

Figure S5: TRPL dataset of BA-MA RP perovskites from n=1 (a) to n=5 (e) across four different excitation powers in the power range.

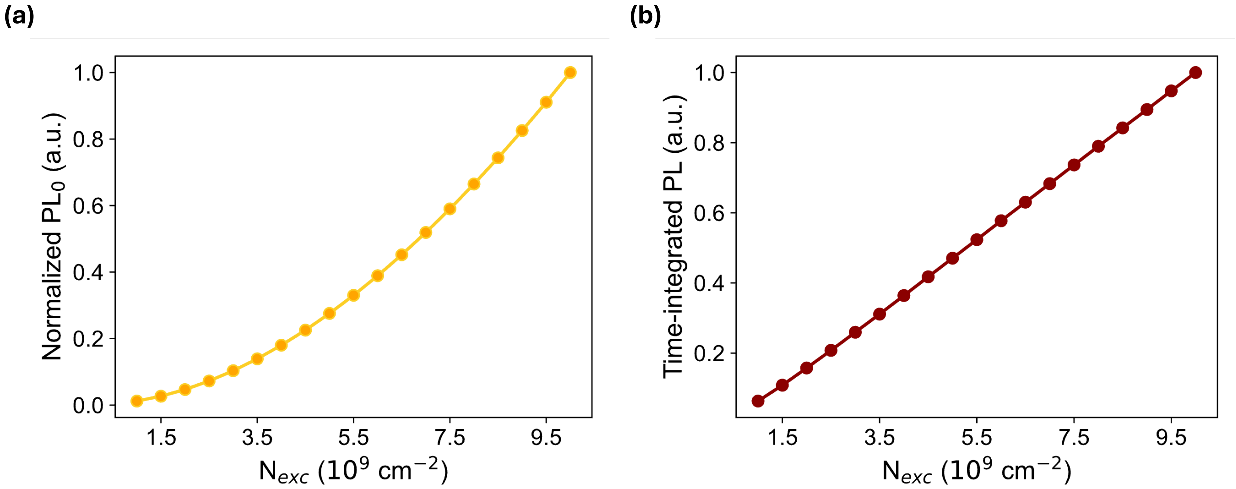

Figure S6: Simulation for free charges carriers (x = 1) and PLQY = 1 of the normalized peak intensity (PL_0) (on the left) and Time-integrated photoluminescence intensity (on the right).

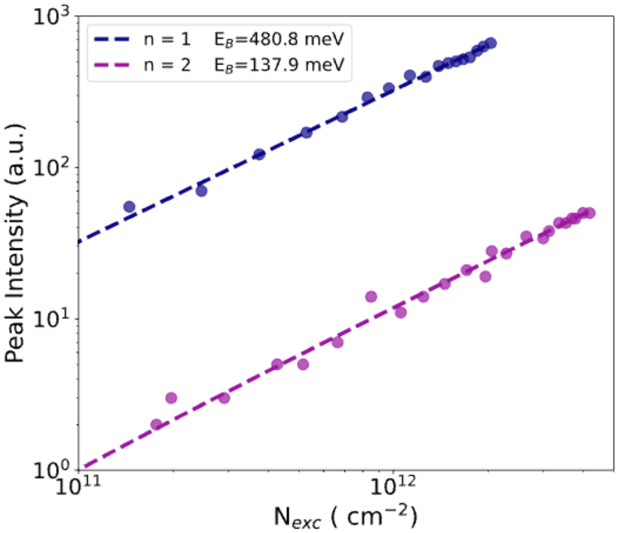

Figure S7: Fitting of equation (5), to the TRPL experiments of PEA-FA RP perovskites n=1,2. The dots represent the experimental data, while the dashed lines correspond to the fits.

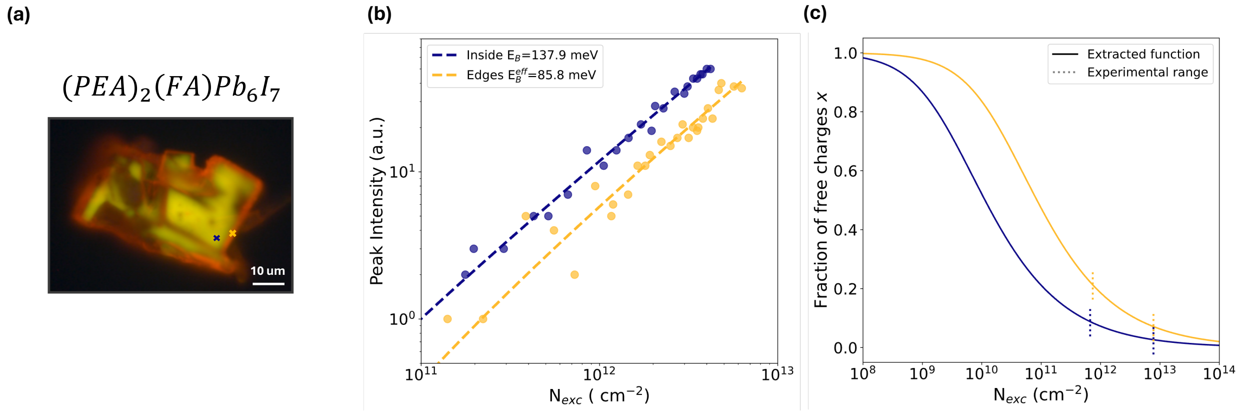

Figure S8: (a) Reflectivity image of $PEA_2FAPb_6I_7$. The marker (x) indicates the two regions where the time resolved photoluminescence (trpl) experiments were performed. (b) Fitting of equation (5) to the trpl experiments. The dots represent the experimental data, while the dashed lines correspond to the fits. (c) Fraction of free charges x calculated using the fit results, extended over the range 10^8 cm^{-2} $< N_{exc} < 10^{14}$ cm^{-2}.

Theoretical model

In materials without a significant number of trap states, free exciton populations $N(t)$ obey the following differential equation:

$$\frac{dN(t)}{dt} = -(\nu + \phi)N(t) \tag{2}$$

where ν and ϕ stand for the radiative and non-radiative decay rates. The solution of this equation is just the typical exponential decay.

$$N(t) = N_0 e^{-(\nu+\phi)t} \tag{3}$$

In a small interval $[t,\ t + dt]$, the number of photoluminescence photons emitted should be expressed as $E(t) = \nu N(t)dt$, with the peak emission $E = \nu N_0 dt$ occuring at the time of the incident laser pulse, $t = 0$. This linear relation corresponds to the first order recombination process, the excitonic case. However, when an excitation creates an electron-hole pair, it might not remain bound together. If charges diffuse freely, then the rates of recombination become proportional to the product of the concentrations of electrons and holes, as the reaction takes place when opposite charges encounter each other. If we assume that electrons and holes diffuse at the same rate, then the same function represents the concentrations of both species. The peak photoluminescent emission would be proportional to the square of the concentration $E = \rho N_0^2 dt$.

In systems in which both excitons and free carriers are present, the peak photoluminescent emission equals:[9]

$$E = \left(\rho x^2 N_0^2 + \nu(1 - x)N_0\right)\, dt, \tag{4}$$

where x represents the fraction of pair excitations in the form of free charges (as opposed to excitons). Here we have assumed that we can disregard Auger recombination. This mixed case is generally treated in the literature only at a qualitative level, typically by

employing a power-law fit that yields an exponent β between 1 and 2. Starting from a statistical description of excitons and free charges, however, it is possible to derive the a two-dimensional analogue of the Saha equation[10] for a system of excitons and free charges.

We begin by distinguishing two cases in the energy. For a pair of free charges (exciton and hole) the energy equals the sum of their kinetic and interaction energies, but when they move independently of each other, we can approximate the energy using only the kinetic energy part.

$$H_{\text{free}} = \frac{p_e^2}{2\mu_e} + \frac{p_h^2}{2\mu_h}.$$

When they are bound as an exciton, the energy equals the kinetic energy of the exciton plus the binding energy E_b:

$$H_{\text{bound}} = \frac{p_{\text{exc}}^2}{2\mu_{\text{exc}}} - E_b.$$

The probability of finding the pair of charges in a given state on a two-dimensional layer equals:

$$p = \frac{1}{Zh^4} e^{-\beta H}$$

where $\beta = \frac{1}{k_B T}$ and H stands either for H_{free} or H_{bound} depending on the configuration considered. The partition function Z equals the sum (or integral) over all the admissible values of the position and momenta of $\exp(-\beta H)$, and may be thought of as a normalization factor for the probabilities. When we think of the pair of charges as a single exciton, though, we must replace the h^4 in the denominator by an h^2, corresponding to the fact that we are dealing with a single particle and not two.

To calculate the probability of being in any free state, we integrate p for all the values of position and momenta for which the energy is H_{free} and obtain

$$p_{\text{free}} = \frac{1}{Z} \int p \, dp_x \, dp_y \, dx \, dy = \frac{1}{Z} \frac{(2\pi k_B T S)^2}{h^4} \, \mu_e \mu_h,$$

with S the total area in the system. If we wish to know the probability of finding both

charges bound together as an exciton, we integrate over all the bound states and get:

$$p_b = \frac{1}{Z}\,\frac{2\pi k_B T S}{h^2}\mu_b e^{E_b/(k_B T)},$$

where μ_b would be the effective mass of the exciton.

The ratio of these two probabilities equals

$$\frac{p_f}{p_b} = \frac{2\pi k_B T S}{h^2}\,\frac{\mu_e \mu_h}{\mu_b} e^{-E_b/(k_B T)}.$$

Suppose we create a large number N_0 of pair excitations. Then the number of these pairs that are excitons at equilibrium will equal $N_b = N_0 p_b$. For such large numbers of particles, we can approximate the probability of finding a given pair of opposite charges in a free state by treating the probability of each charge as independent. In other words, the probability that two randomly-picked opposite charges are free equals the probability of the electron being free times the probability of the hole being free $p_f = p_{f,e}\ p_{f,h}$. We will assume that $p_{f,e} = p_{f,h} = x$, and that $p_b = 1 - x$.

Another important point to bear in mind concerns the probability p_b. When we create N_0 excitation pairs, an electron may form an exciton with any of the N_0 holes in the system (the many-body energy H would contain N_0^2 interaction energy terms). Combining these insights, we arrive at the desired Saha equation for our system.

$$\frac{x^2}{1-x} = \frac{1}{n_0}\frac{2\pi \mu k_B T}{h^2} e^{-E_b/(k_B T)},$$

with $n_0 = N_0/S$ the surface concentration of excitations, and $\mu = \mu_e \mu_h / \mu_b$.

Resolving the equation for x, the photon emission intensity for a carrier concentration $\tilde{n}$ can be expressed as follows:

$$E = (\rho x^2 \tilde{n}^2 + \nu(1-x)\tilde{n})\Delta t = (\rho + \nu)\left(\tilde{n} + \frac{1}{2} - \sqrt{\tilde{n} + \frac{1}{4}}\right)\Delta t \tag{5}$$

yielding an expression for the photoluminescence intensity E_0 that explicitly captures the dependence of the free-carrier fraction on the excitation density. We can define the local (concentration-dependent) exponent $\beta(\tilde{n})$ by calculating the slope.

The function $\beta(\tilde{n})$ decreases monotonically, and we can immediately see that the two limits go to 1 for $\tilde{n} \to \infty$ and to 2 $\tilde{n} \to 0$:

$$\lim_{\tilde{n}\to\infty} \beta(\tilde{n}) = 1$$

$$\lim_{\tilde{n}\to 0} \beta(\tilde{n}) = \lim_{\tilde{n}\to 0} \left[1 + \frac{1}{2\tilde{n}}\left(\sqrt{4\tilde{n}+1} - (2\tilde{n}+1)\right)\right] = 2$$

The fit parameter of our theory is the Saha prefactor A, which contains E_b, μ , T and other physical constants (k_B, h). Fixing the μ (that in the case of RPs perovskite is around $0.2m_0$[11]), and assuming ambient temperature, we can derive the exciton binding energy E_b (although, being in an exponent, the result may be very sensitive to small errors in the measurements[†]). Table S1 reports the extracted values for E_b and the extracted free charges fraction at solar fluence ($N_{exc} \sim 10^{10}$ cm^{-2}).

n	RPPs	μ^* (units of m_0)	E_b (meV)	Fraction of free charges at solar fluence ($N_{exc} \sim 10^{10}$ cm^{-2}) (extracted)
1	$(BA)_2PbI_4$	0.221	430 ± 20	0
2	$(BA)_2(MA)Pb_2I_7$	0.217	139 ± 9	0.51 ± 0.06
3	$(BA)_2(MA)_2Pb_3I_10$	0.201	113 ± 13	0.67 ± 0.08
4	$(BA)_2(MA)_3Pb_4I_13$	0.196	80 ± 5	0.85 ± 0.02
5	$(BA)_2(MA)_4Pb_5I_16$	0.186	59 ± 8	0.92 ± 0.02

Table S1: Excitation properties in RPs derived from the experimental data. *Data from Blancon *et al.*[11] E_b and fraction of free charges are extracted using our presented work.

Having determined the exciton binding energy for each number of layers n, we can simulate the decay of the population of charge pairs $N(t)$ with time by numerically solving the

[†]To calculate the error for $n = 1$, we start from the fit of the experimental data to Eq. (5), which yields a 200% relative error for the A. This implies an error in the energy in the exponent that might lead to a change in A of up to a factor of 2, or $\exp(-\Delta E_b/(k_B T)) \approx 2$. Consequently, we can estimate that the error in the binding energy: $\Delta E_b \approx \ln(2)\ k_B T \approx 20$ meV.

following differential equation:

$$\frac{dN}{dt} = -N\ (\nu\ (1-x) + \rho x N), \tag{6}$$

which represents the change in the population number due to exciton recombination (at a rate ν) and free charge recombination (that occurs with a frequency determined by the constant ρ). At each time step, we use the Saha equation to recalculate the fraction x using the population at that time. We can fit the ν and ρ parameters by comparing the numerical results to experimental data, remembering that exciton and free charge recombinations might have different quantum yields. The relative quantum yield therefore implies an extra fitting parameter q_r.

The numerical predictions agree with the experimental data for reasonable values of the recombination rates, assuming a fixed relative quantum yield q_r (see Table S2 and Figure S9). Figure S10 reports the numerical prediction at solar fluence ($N_{exc} = 10^{10}\mathrm{cm}^{-2}$). The results are normalised to the peak PL intensity.

n	ν [ns^{-1}]	ρ [ns^{-1}]
1	3.00	3×10^{-3}
2	0.15	3×10^{-3}
3	0.25	3×10^{-3}
4	10^{-3}	1.5×10^{-3}
5	0.5×10^{-3}	0.75×10^{-3}

Table S2: Parameter values for the numerical evolution of the population of charge pairs $N(t)$ in time. relative quantum yield for excitons compared to free charges was set to $q_r = 0.2$.

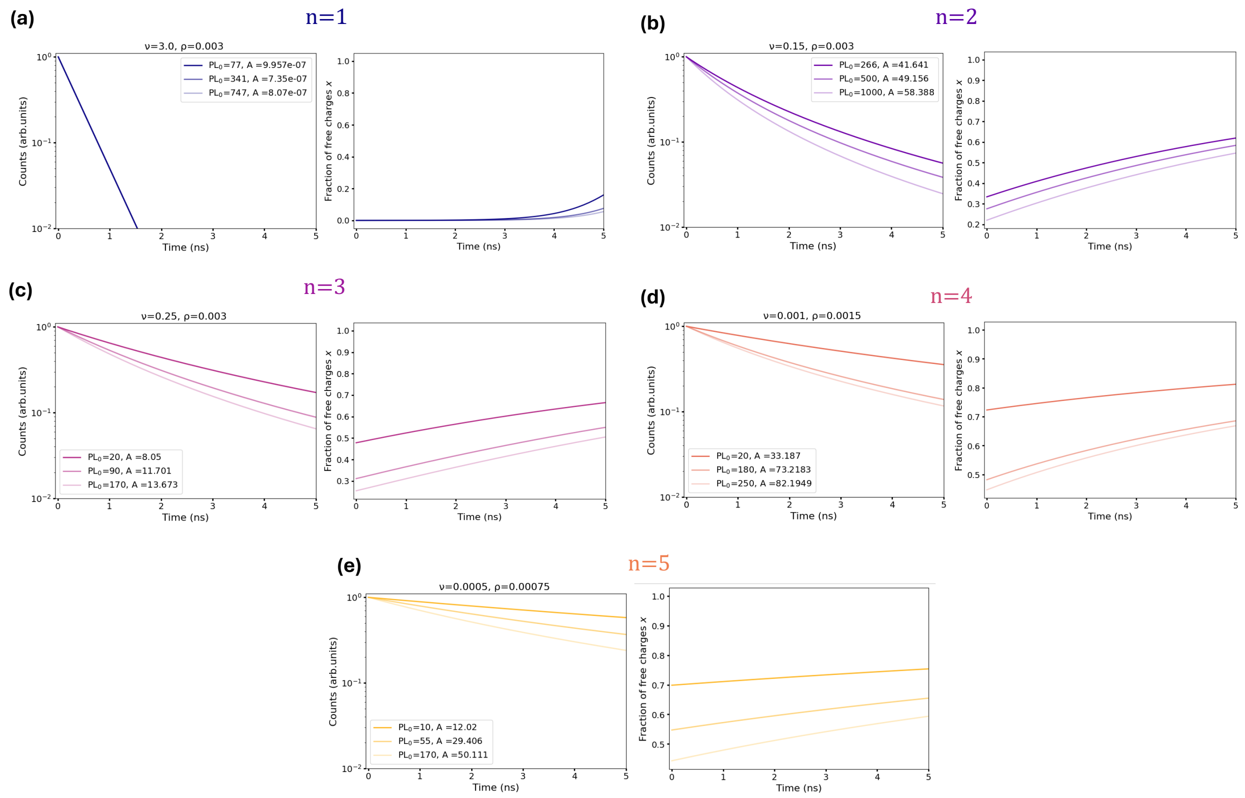

Figure S9: (a-e) TRPL simulations and corresponding fraction of free charges x at different excitation fluencies for $n = 1, 2, 3, 4, 5$ using the determined exciton binding energy (Table S1). The reported value of A in the simulations was chosen to match the same $\tilde{n} = N_{exc}/A$ and the same peak intensity at $t = 0$ (PL_0) as in the experimental measurements.

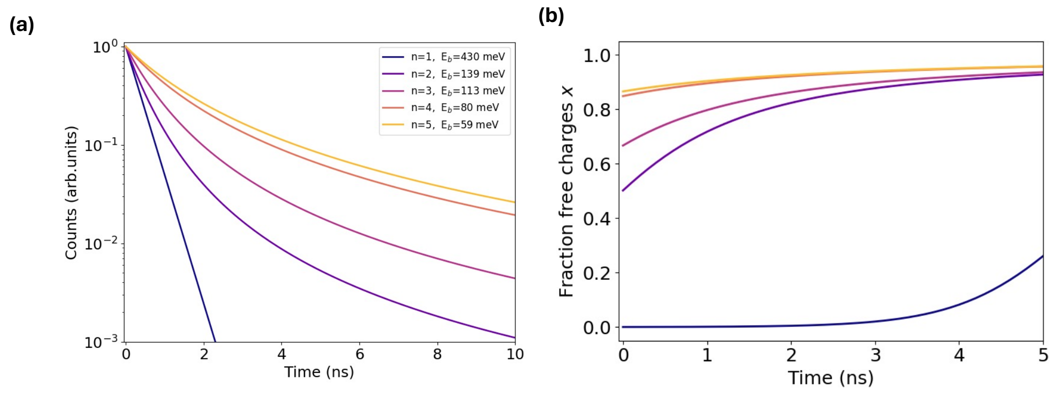

Figure S10: TRPL simulations (a) and corresponding fraction of free charges x (b) at solar fluence for $n = 1, 2, 3, 4, 5$. The parameters values for ν, ρ, and q_r were set according to Table S2.

References

(1) Stoumpos, C. C.; Cao, D. H.; Clark, D. J.; Young, J.; Rondinelli, J. M.; Jang, J. I.; Hupp, J. T.; Kanatzidis, M. G. Ruddlesden–Popper Hybrid Lead Iodide Perovskite 2D Homologous Semiconductors. *Chemistry of Materials* **2016**, *28*, DOI: 10.1021/acs.chemmater.6b00847.

(2) Di, J.; Chang, J.; Liu, S. (Recent progress of two-dimensional lead halide perovskite single crystals: Crystal growth, physical properties, and device applications. *EcoMat* **2020**, *2*, DOI: 10.1002/eom2.12036.

(3) La-Placa, M.-G.; Guo, D.; Gil-Escrig, L.; Palazon, F.; Sessolo, M.; Bolink, H. J. Dual-source vacuum deposition of pure and mixed halide 2D perovskites: thin film characterization and processing guidelines. *Journal of Materials Chemistry C* **2020**, *8*, DOI: 10.1039/c9tc06662d.

(4) Kim, K.; Park, C.; Cha, E.; Kang, D.; Park, J.; Cho, S.; Yi, Y.; Park, S. Impact of light illumination on the surface structure of two-dimensional Ruddlesden–Popper perovskite in the fabrication process. *Journal of Physics: Energy* **2023**, *5*, DOI: 10.1088/2515-7655/acc2e6.

(5) Liang, Y.; Liu, F.; Xie, X.; Ma, Y.; Guan, Y.; Yu, W.; Zou, Y.; Zhang, L.; Zhang, X.; Zhang, Y.; Li, B.; Wu, C.; Jiang, K.; Xiao, L.; Zou, D.; Zheng, S. Realizing Phase-Pure $(PEA)_2FAPb_6I_7$ Perovskite Films by Inhibiting 3D Phase Formation. *Advanced Functional Materials* **2024**, *34*, DOI: 10.1002/adfm.202401257.

(6) Stoumpos, C. C.; Cao, D. H.; Clark, D. J.; Young, J.; Rondinelli, J. M.; Jang, J. I.; Hupp, J. T.; Kanatzidis, M. G. Ruddlesden–Popper Hybrid Lead Iodide Perovskite 2D Homologous Semiconductors. *Chemistry of Materials* **2016**, *28*, DOI: 10.1021/acs.chemmater.6b00847.

(7) Delport, G.; Chehade, G.; Lédée, F.; Diab, H.; Milesi-Brault, C.; Trippé-Allard, G.; Even, J.; Lauret, J.-S.; Deleporte, E.; Garrot, D. Exciton–Exciton Annihilation in Two-Dimensional Halide Perovskites at Room Temperature. *The Journal of Physical Chemistry Letters* **2019**, *10*, DOI: 10.1021/acs.jpclett.9b01595.

(8) Spitha, N.; Kohler, D. D.; Hautzinger, M. P.; Li, J.; Jin, S.; Wright, J. C. Discerning between Exciton and Free-Carrier Behaviors in Ruddlesden–Popper Perovskite Quantum Wells through Kinetic Modeling of Photoluminescence Dynamics. *The Journal of Physical Chemistry C* **2020**, *124*, DOI: 10.1021/acs.jpcc.0c06345.

(9) Nagaya Wong, N.; Ha, S. K.; Williams, K.; Shcherbakov-Wu, W.; Swan, J. W.; Tisdale, W. A. Robust estimation of charge carrier diffusivity using transient photoluminescence microscopy. *The Journal of Chemical Physics* **2022**, *157*, DOI: 10.1063/5.0100075.

(10) Saha, M. N. On a physical theory of stellar spectra. *Proceedings of the Royal Society of London. Series A, Containing Papers of a Mathematical and Physical Character* **1921**, *99*.

(11) Blancon, J.-C.; Stier, A. V.; Tsai, H.; Nie, W.; Stoumpos, C. C.; Traoré, B.; Pedesseau, L.; Kepenekian, M.; Katsutani, F.; Noe, G. T.; Kono, J.; Tretiak, S.; Crooker, S. A.; Katan, C.; Kanatzidis, M. G.; Crochet, J. J.; Even, J.; Mohite, A. D. Scaling law for excitons in 2D perovskite quantum wells. *Nature Communications* **2018**, *9*, DOI: 10.1038/s41467-018-04659-x.
